# Opportunities and Challenges for Machine Learning in Rare Diseases

**DOI:** 10.3389/fmed.2021.747612

**Published:** 2021-10-05

**Authors:** Sergio Decherchi, Elena Pedrini, Marina Mordenti, Andrea Cavalli, Luca Sangiorgi

**Affiliations:** ^1^Computational and Chemical Biology, Fondazione Istituto Italiano di Tecnologia, Genoa, Italy; ^2^Department of Rare Skeletal Disorders, IRCCS Istituto Ortopedico Rizzoli, Bologna, Italy; ^3^Department of Pharmacy and Biotechnology (FaBiT), Alma Mater Studiorum – University of Bologna, Bologna, Italy

**Keywords:** machine learning, rare disease, disease registry, open data, clinical decision support system

## Abstract

Rare diseases (RDs) are complicated health conditions that are difficult to be managed at several levels. The scarcity of available data chiefly determines an intricate scenario even for experts and specialized clinicians, which in turn leads to the so called “diagnostic odyssey” for the patient. This situation calls for innovative solutions to support the decision process *via* quantitative and automated tools. Machine learning brings to the stage a wealth of powerful inference methods; however, matching the health conditions with advanced statistical techniques raises methodological, technological, and even ethical issues. In this contribution, we critically point to the specificities of the dialog of rare diseases with machine learning techniques concentrating on the key steps and challenges that may hamper or create actionable knowledge and value for the patient together with some on-field methodological suggestions and considerations.

## Introduction

A rare disease (RD) is defined as a low-prevalence condition that affects fewer than one in 2,000 people. Due to the frequent lack of knowledge and treatment (which makes them also known as “orphan diseases”), they represent a real emerging global public health priority. So far 6,000–7,000 distinct RDs have been recognized, affecting 4–6% of the European population, and 300 million persons globally (1). From a clinical perspective, RDs are extremely heterogeneous and complex, often characterized by different clinical subtypes and overlapping phenotypic manifestations. Although most of the RDs are classified as “genetic diseases,” (2, 3) the causes remain unclear for many of them, making the identification of therapies troublesome.

Different from other clinical fields, RDs are often lacking specific and adequate public health policies and can be considered as a real health system challenge. Difficult and delayed diagnosis (with diagnostic processes taking many years and unnecessary costs), unknown molecular mechanisms, lack of specific treatments, and scattered patient data are all responsible for the difficulty in both taking care of these patients and setting up research activities. This makes RDs a major public health problem, and many challenges hamper the development of therapies. In addition, they are often neglected by major public and industrial funding with a limited interest of pharmaceutical companies (4, 5).

Overall, RDs are responsible for enormous healthcare costs, just for the difficulties in diagnosis and their often serious health degenerative consequences. To reduce RDs healthcare costs and to optimize the assistance of patients, new effective treatments are required, making it necessary to promote research with new strategies. Recent advances in next-generation sequencing (NGS) have already represented a great opportunity (6); in particular, whole exome or whole genome approaches have strongly improved the diagnosis and shortened the “diagnostic odyssey” (7), also helping in the molecular characterization of diseases. Data coming from many other innovative technologies such as advanced imaging techniques, multiomics, gait analyses, and others (depending on the clinical field) represent an invaluable source of information too. As a result of all these new approaches and technologies, there is a huge amount of available data (never collected before) to be managed and analyzed according to privacy regulations, still with a limited sample set (number of patients). This scenario is a big data one in the omics component, but not in terms of the sample size.

As an innovative discipline for data modeling, machine learning (ML) is becoming a great opportunity. ML is a branch of artificial intelligence (AI) rooted in statistics that learns from data (the examples) and then performs predictions on new unseen data. By using specific algorithms, and typically large datasets, the goal is to use available data to make classifications or predictions in general, uncovering not previously discovered key insights, which will potentially drive the decision on the diagnosis and treatment options of a patient.

**Figure 1:**
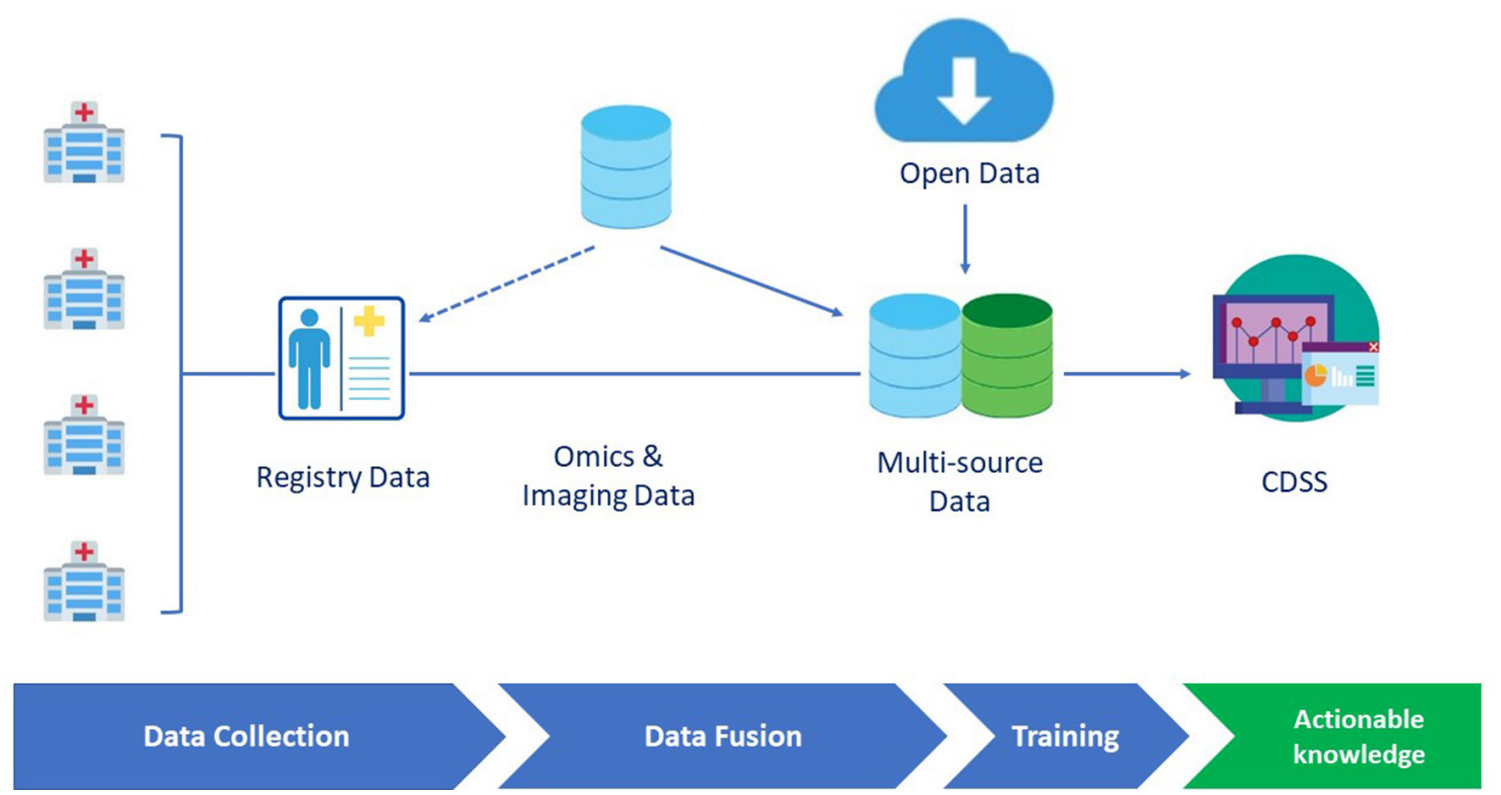
A prototypical data flow pipeline in the clinical decision support system (CDSS) dedicated to rare diseases (RDs). Omics and imaging data can either be integrated from different sources or be collected as part of disease registry data. Data are then fed to the learning engine and the results are provided through a CDSS GUI interface.

During the last two decades, AI and ML have been characterized by an unprecedented development, also supported by empowered computational means (i.e., graphical processing units). However, to further improve their applicability in healthcare challenges, it is essential to consider the compatibility of RDs specificities with respect to ML approaches. In the following, we critically discuss the role of the two key ingredients of any ML attempt namely, the data and the methods (and their interplay) (8). We discuss in detail diseases registries, genuinely public datasets, and lastly, methodological approaches, and ML challenges for RDs. Figure 1 summarizes a prototypical pipeline for the data flow in a clinical decision support system.

## Disease Registries

By definition, a registry is “an organized system that uses observational study methods to collect uniform data (clinical and other) to evaluate specified outcomes for a population defined by a particular disease, condition, or exposure, and that serves one or more predetermined scientific, clinical or policy purposes” (9). Among different registries, the disease registry represents the pivotal tool in supporting RD research and care, since the primary aims are collection, analysis, and dissemination of information on a group of people defined by a particular disease (10).

Many stakeholders recognize the crucial role of a high-quality registry and uniformity in data collection, particularly for networking activities. In 2015, the European Medicines Agency has established a patient registry initiative to promote registry data collection and reuse for postauthorization safety study and postauthorization effectiveness study (11). Moreover, 24 European Reference Networks (ERNs) [wanted by the European Commission (EC)] were installed in 2017 to facilitate the discussion on complex or RDs that require highly specialized treatment and concentrated knowledge (2014/286/EU). The EC defined specific criteria for ERNs, encouraging the research and epidemiological surveillance through shared patient registries (12–14).

Nonetheless, the RDs domain may greatly benefit from data pooling, since information on orphan patients is frequently scattered across different hospitals and institutions (14, 15). To promote the merging of standardized data, the European Rare Diseases Platform has released the “Set of common data elements for Rare Diseases Registration” produced by a Working Group coordinated by the Joint Research Center. In addition, the semantic compatibility of phenotypic data captured within a registry can be ensured by the implementation of ontologies, standards, and dictionaries, like Human Phenotype Ontology (16) and ORPHAcode. The process to make registry data findable, accessible, interoperable, and reusable (FAIR) surely increases the quality of information, but at the same time enhances the potential extensive use of the captured data to improve research and to promote patient health. The FAIR principles allow data sharing, including tools and workflows, from different registries using the same syntax (12, 17, 18).

Moreover, some legal and ethical obstacles can afflict data pooling, restricting the range of action of the registry. The sharing of personal and clinical data, even pseudonymized, presents privacy issues. The European General Data Privacy Regulation (GDPR; EU Regulation 2016/679) allows data-free movement, even if the sensitive nature of phenotypic information requires a rigorous balancing between data protection to avoid mistreatment and the data accessibility to promote accurate research networking activities. Accordingly, a solid framework that addresses privacy issues and ethical and social implications becomes mandatory.

All the mentioned approaches, put in place to pursue the establishment of a high-quality disease registry, were the grounds on which our group has created and implemented five RD registries. These registries realized aiming both care and research purposes, address four skeletal orphan disorders (Multiple Osteochondromas, Osteogenesis Imperfecta, Ollier-Maffucci Diseases, Ehlers-Danlos syndrome), and one oncological rare condition (Li-Fraumeni syndrome). All of them rely on a web-based platform, genotype-phenotype data integration (GeDI) platform, established on a relational database. GeDI was created considering the JRC “Set of common data elements for Rare Diseases Registration,” as well as highly recommended ontologies (HPO, ORPHAcode, HGVS, and ICF), and following GDPR and privacy requirements.

Until a few years ago, the phenotypic information was not considered big (19), but with the evolution in terms of standardization and FAIRness, the consequent simplification in data merged across healthcare providers, and the integration among different data sources transformed clinical data into new types of big files. The primary and essential investigation of skeletal disorders is imaging data, ranging from traditional X-Rays and ultrasounds, through hybrid imaging such as positron emission tomography/MRI (PET/MRI) up to innovative instruments like high-resolution peripheral quantitative CT (HR-pQCT) (20). These data are increasingly needed to support the diagnostic process, to longitudinally follow-up disease evolution, and to promote translational research. The integration of imaging data with all other detailed phenotypic information is becoming mandatory to obtain a complete overview of patient manifestations. Similarly, the rapid advancement in NGS approaches and the parallel explosion of bioinformatics has revolutionized the research on RDs, reinforcing the understanding of biological pathways and pathomechanisms (21, 22). The accompaniment of NGS and imaging data to deep phenotyping is a fundamental enrichment for rare skeletal disease research. The analysis of that notable amount of data requires *ad hoc* computational solutions, like ML approaches (23).

The rarity of orphan patients, despite the presence of registries, still has an impact on ML analyses highly, hence then open data can highly contribute to support the modeling attempt.

## Open Data

As clearly stated by Cohen, “medical artificial intelligence is particularly data-hungry” (24); nonetheless, the demand is limited by the reduced availability of trusted and reliable biomedical data (25). Public or open datasets must respond to three main criteria: online availability, the absence of costs, and reusability (26). Public data may represent a solution, considering that they create value in multiple heterogeneous areas (healthcare, city security, savings, etc.); therefore, numerous worldwide countries have implemented governmental open data sites (27) to increase findability and accessibility.

The open data role in biomedical research is widely recognized and scientists boost public sharing of resources at an increasing speed. Free access to data would expedite research and open new opportunities in scientific research, improving care and treatment; nevertheless, some substantial pitfalls and issues still exist (28).

The first limitation is represented by the lack of harmonization principles governing data (28) and the presence of multiple standards is a known concern on data sharing and biomedical information reuse (23). Common “languages,” in terms of formats and ontologies, are continually being improved for innovative data types (i.e., omics), but compatibility among sources is affected by the variability of standards (when present) on many other data elements and related metadata (i.e., phenotyping) (23).

Another challenging point is the reliability of public data (29). This aspect can include a variety of subtopics that carries costs, like the unavailability of ongoing quality control, the lack of updating of datasets, the absence of support for potential users and the need of highly specialized human resources.

The final and critical point is the use of open data for rare conditions. This peculiar scenario amplifies the aforementioned concerns. At the same time, the need for public data is clear in paving the way for prompt diagnosis, innovative treatments, personalized care, and research activities (23).

## Machine Learning for Rare Diseases

Machine learning, as already anticipated, is a wide and largely heterogeneous subfield of computer science that in the last 20 years has evolved toward a consolidated and largely useful discipline. In ML, one is interested in building a robust and predictive model, which for instance, within a certain degree of accuracy, can predict a class (classification) or find patterns on data (i.e., groups *via* clustering). In the first case, when applied to clinical data, one often talks of diagnosis prediction, and in the second, it is often about the stratification of patients. Many other learning paradigms are available and despite the ubiquitous success achieved in many applications ranging from engineering problems to the life science, the systematic application of ML methods to clinical practice is still relatively modest albeit starting to be present in clinical decisions support systems (30–33). There are many reasons that hamper the widespread diffusion of ML in the clinic, and in the case of RDs, this scenario is amplified by several specificities (34, 35), which, however, the scientific community is addressing *via* methods, protocols, and technologies in general.

In the RD, the most important limitation in building meaningful predictive models, either supervised or agnostic, with respect to *a priori* labeling, is the data collection process (36–38). Although deep learning models typically require thousands of samples to converge to robust solutions, shallow (i.e., limited parameters) models still require in the order of hundreds of samples to build acceptably robust models. It should be stressed that ML aims at building predictive models, in other words, models that can be used out-of-sample. While it could be considered sufficient for a qualitative analysis having a limited sample size and/or finding associations as in epidemiological studies (39), in the ML case, there is a more ambitious modeling attempt that is deemed to fail if working in a very restrictive small sample regime.

To deal with the small sample regime, the machine literature provides several possibilities: one may use available data possibly extending the collection outside the disease of interest to collect unlabeled examples (40–42), one can inherit from similar models [transfer learning (43)] to just fine-tune the model and lastly, one could even imagine a data augmentation strategy (44), that is finding ways to populate the dataset with new artificially built samples.

The strategy of collecting more unlabeled data is widely applicable as it requires gathering more data from possibly more controls or even more from diseases different from the current disease under analysis. This is particularly relevant for rare diseases where many patients with “uncertain” diagnosis can be present; collecting this additional unlabeled data can give interesting information about the manifold where data live.

On the other side, data augmentation, possibly through ingenious generative techniques (45), can be another original way to face the data scarcity problem. In this second case, however, it is more difficult to assess the reliability of this modeling. First, generative networks often need a large amount of data to be trained, and second, inferring new data based on a manifold implicitly learned on few data may lead to a partial tautology rendering the overall strategy perilous.

The small sample regime, despite being probably the most impactful and first problem to be faced when dealing with the RD field, is not however the only point to be carefully addressed when modeling such data. Despite the wide success of deep learning paradigms in big data scenarios (to be precise big sample sizes), they often deliver not easily interpretable models. To allow the clinician to understand the meaning of a classification result, it is, therefore, necessary to resort to possibly less complex but explainable models (46). At a technical level, this brings to the scene chiefly linear models [possibly sparse ones (47)] and non-linear rule-based models, such as decision trees (48) or switching neural networks (49) for instance.

The availability and effectiveness of explainable models still are necessary, but not sufficient conditions to determine robust and explainable models. Indeed, explainable models are valuable when the explanation that they deliver is stable and robust inside the domain they deal with (assuming the same learning method) and across learning algorithms, ideally. This means that feature weighting/extraction must be a stable process to allow the clinician to get a value from the obtained results; this far from the trivial problem is feature stability, something we recently discussed for epidemiological data (50). Albeit often neglected in practice, this problem is relevant particularly when coupled with the small regime of RDs (50) and sample sizes in general; unsupervised feature selection techniques can mitigate this issue (50).

However, when dealing with features it can be relevant to consider the fairness (51). In other words, when determining a disease condition possibly “confounding” factors, such as gender and social status should be protected features, that is *a priori* one postulates that the gender or another feature to be protected cannot determine the disease (or any other) outcome. While this view today is quite uncommon, yet in clinical ML, for sensitive disease or particular case-dependent conditions could be of utility and necessary to protect specific patient characteristics to avoid discriminations and exacerbate iniquities.

Feature sets (clinical or omics) are associated inevitably with costs and time. Getting an X-ray is different from other diagnostic tools, possibly not standard, such as for instance, collective lipidomic signatures through mass Spectrometry (52). As these features set links with different time and cost profiles, feature selection is particularly challenging as one would like to maintain the representation power of possibly costly non-standard features, while at the same time maintaining a fast and inexpensive diagnostic tool. These contrasting forces together with, again, the small data regime call for proper solutions that allow obtaining a quantitative compromise (a multiobjective optimization problem) between accuracy, explainability, and cost/time effectiveness of the selected clinical or omics data necessary for diagnosis.

It is hence evident that the delivery of knowledge has several, sometimes tight, prerequisites which if not met cannot allow any meaningful analysis; while methods development is fundamental to the ML field, it is tantamount clear that in clinical ML for RD the data is the undiscussed protagonist.

A last key aspect is the privacy preserving issue that, for Europe, translates into GDPR compliance, as already mentioned. Historically, ML methodologies have been devised having in mind all the data resident in the same local storage; this is something largely unmet by the clinical reality where each hospital/research center has its own dataset/registry not in sync typically with a central shared, common repository. This situation is the absolute standard for clinical ML and RDs share this liability. The need to maintain privacy and avoiding to move a significant amount of data inspired what is now commonly under the name of federated ML (53). In this learning paradigm, data is resident on the original data infrastructure and on the network, only parameters are shared. Federated ML requires a specific rethinking of algorithms; this is a beneficial stimulus to the community, but still requires both a theoretical and programming effort to redesign and reimplement theoretically sound and well-established mathematical methodologies. It is promising that for instance in Europe, this need for federation has been largely and overall correctly perceived by the policymaker through initiatives like Gaia-X (54) which have the specific objective of creating a trustable, distributed, and federated data sharing infrastructure. Interestingly, very recently, the Swarm distributed learning paradigm (55) has been pushed as a further development of federated learning, offering the explicit capability of nodes of avoiding relying on a central repository of learning parameters, thus creating an effective collective swarm of collaborating agents. This technology also involves decentralized data structures as the blockchain and represents a very interesting protocol to deal safely with privacy concerns.

## Conclusions

In this contribution, we have discussed what ML has to deal with in trying to effectively face the RD issue to grant robust, usable, and actionable knowledge to the clinician. While several points are shared with the more general realm of the clinical machine learning, RDs pose specific challenges and for instance, present an unusual big data regime, in which one has potentially a huge omics data but still for a limited number of patients, thus bringing the typical bioinformatics scenario of several features, small samples. The proof-of-time of ML solutions will have to deal with the discussed specificities, and the solution is inevitably a well-concerted mix of rigorous math, trusted and privacy preserving technologies, and chiefly standardization for data curation and federation.

## Author Contributions

SD, EP, and MM contributed to conception and design of this mini-review. They were deeply involved in all the steps of the manuscript preparation, references collection and evaluation of the contest. They have drafted the article and participated in all the steps of its revising. AC has participated to paper design and supported the other authors in structuring the manuscript. He has critically revised the manuscript during the drafting process. LS has contributed to conception and design of this mini-review and he supported all the activities. He has participated step by step at the revising process with concrete suggestions and integrations. All the authors approved the final version for publication and agreed to be accountable for all aspects of the work in ensuring that questions related to the accuracy or integrity of any part of the work are appropriately investigated and resolved.

## Conflict of Interest

The authors declare that the research was conducted in the absence of any commercial or financial relationships that could be construed as a potential conflict of interest.

## Publisher's Note

All claims expressed in this article are solely those of the authors and do not necessarily represent those of their affiliated organizations, or those of the publisher, the editors and the reviewers. Any product that may be evaluated in this article, or claim that may be made by its manufacturer, is not guaranteed or endorsed by the publisher.

## References

[1] Nguengang Wakap S Lambert DM Olry A Rodwell C Gueydan C Lanneau V. Estimating cumulative point prevalence of rare diseases: analysis of the orphanet database. Eur J Hum Genet. (2020) 28:165–73. 10.1038/s41431-019-0508-0 31527858 PMC6974615

[2] Sernadela P González-Castro L Carta C van der Horst E Lopes P Kaliyaperumal R. Linked registries: connecting rare diseases patient registries through a semantic web layer. Biomed Res Int. (2017) 2017:8327980. 10.1155/2017/8327980 29214177 PMC5682045

[3] Ekins S. Industrializing rare disease therapy discovery and development. Nat Biotechnol. (2017) 35:117–8. 10.1038/nbt.3787 28178258 PMC5320585

[4] Stoller JK. The challenge of rare diseases. Chest. (2018) 153:1309–14. 10.1016/j.chest.2017.12.018 29325986

[5] Ahmed MA Okour M Brundage R Kartha RV. Orphan drug development: the increasing role of clinical pharmacology. J Pharmacokinet Pharmacodyn. (2019) 46:395–409. 10.1007/s10928-019-09646-3 31338634

[6] Fernandez-Marmiesse A Gouveia S Couce ML. NGS technologies as a turning point in rare disease research, diagnosis and treatment. Curr Med Chem. (2018) 25:404–32. 10.2174/0929867324666170718101946 28721829 PMC5815091

[7] Boycott KM Hartley T Biesecker LG Gibbs RA Innes AM Riess O. A diagnosis for all rare genetic diseases: the horizon and the next frontiers. Cell. (2019) 177:32–7. 10.1016/j.cell.2019.02.040 30901545

[8] Roh Y Heo G Whang SE. A survey on data collection for machine learning: a big data – ai integration perspective. IEEE Transac Knowl Data Eng. (2021) 33:1328–47. 10.1109/TKDE.2019.2946162

[9] Gliklich RE Leavy MB Dreyer NA eds. Registries for Evaluating Patient Outcomes: A User's Guide. 4th ed. Rockville, MD: Agency for Healthcare Research and Quality (US) (2020). 24945055

[10] Zaletel M Kralj M. Methodological guidelines and recommendations for efficient and rational governance of patient registries. National Institute of Public Health. Ljubljana. (2015). 10.1093/eurpub/ckv169.006

[11] McGettigan P Alonso Olmo C Plueschke K Castillon M Nogueras Zondag D Bahri P. Patient registries: an underused resource for medicines evaluation: operational proposals for increasing the use of patient registries in regulatory assessments. Drug Saf . (2019) 42:1343–51. 10.1007/s40264-019-00848-9 31302896 PMC6834729

[12] Kodra Y Weinbach J Posada-de-la-Paz M Coi A Lemonnier SL van Enckevort D. Recommendations for improving the quality of rare disease registries. Int J Environ Res Public Health. (2018) 15:1644. 10.3390/ijerph15081644 30081484 PMC6121483

[13] Ali SR Bryce J Smythe C Hytiris M Priego AL Appelman-Dijkstra NM. Supporting international networks through platforms for tandardized data collection-the European registries for rare endocrine conditions (EuRRECa) model. Endocrine. (2021) 71:555–60. 10.1007/s12020-021-02617-0 33512655 PMC7844549

[14] Opladen T Gleich F Kozich V Scarpa M Martinelli D Schaefer F. U-IMD: the first unified European registry for inherited metabolic diseases. Orphanet J Rare Dis. (2021) 16:95. 10.1186/s13023-021-01726-3 33602304 PMC7893973

[15] Laverty A Jaffé A Cunningham S. Establishment of a web-based registry for rare (orphan) pediatric lung diseases in the United Kingdom: the BPOLD registry [published correction appears in Pediatr Pulmonol. Pediatr Pulmonol. (2008) 43:451–6. 10.1002/ppul.20783 18383113

[16] Köhler S Vasilevsky NA Engelstad M Foster E McMurry J Aymé S. The human phenotype ontology in 2017. Nucleic Acids Res. (2017) 45:D865–76. 10.1093/nar/gkw1039 27899602 PMC5210535

[17] Wilkinson MD Dumontier M Aalbersberg IJ Appleton G Axton M Baak A. The FAIR guiding principles for scientific data management and stewardship [published correction appears in Sci Data. Sci Data. (2016) 3:160018. 10.1038/sdata.2016.18 26978244 PMC4792175

[18] Dos Santos Vieira B Groenen K ‘t Hoen PAC Jacobsen A Roos M Kaliyaperumal R. Applying the FAIR data principles to the registry of vascular anomalies (VASCA). Stud Health Technol Inform. (2020) 271:115–6. 10.3233/SHTI200085 32578552

[19] Delude CM. Deep phenotyping: the details of disease. Nature. (2015) 527:S14–5. 10.1038/527S14a 26536218

[20] Giraudo C Kainberger F Boesen M Trattnig S. Quantitative imaging in inflammatory arthritis: between tradition and innovation. Semin Musculoskelet Radiol. (2020) 24:337–54. 10.1055/s-0040-1708823 32992363

[21] Johnston L Thompson R Turner C Bushby K Lochmuller H Straub V. The impact of integrated omics technologies for patients with rare diseases. Exp Opin Orphan Drugs. (2016) 11:1211–9. 10.1517/21678707.2014.974554

[22] Danielsson K Mun LJ Lordemann A Mao J Lin CH. Next-generation sequencing applied to rare diseases genomics. Expert Rev Mol Diagn. (2014) 14:469–87. 10.1586/14737159.2014.904749 24702023

[23] Rubinstein YR Robinson PN Gahl WA Avillach P Baynam G Cederroth H. The case for open science: rare diseases. JAMIA Open. (2020) 3:472–86. 10.1093/jamiaopen/ooaa030 33426479 PMC7660964

[24] Cohen IG Mello MM. Big data, big tech, and protecting patient privacy. JAMA. (2019) 322:1141–2. 10.1001/jama.2019.11365 31397838

[25] Nielsen SS Krasnik A Rosano A. Registry data for cross-country comparisons of migrants' healthcare utilization in the EU: a survey study of availability and content. BMC Health Serv Res. (2009) 9:210. 10.1186/1472-6963-9-210 19922657 PMC2788548

[26] Chignard S. A Brief History of Open Data. (2013). Available online at: http://parisinnovationreview.com/articles-en/a-brief-history-of-open-data (accessed June 23, 2021).

[27] Kobayashi S Kane TB Paton C. The privacy and security implications of open data in healthcare. Yearb Med Inform. (2018) 27:41–47. 10.1055/s-0038-1641201 29681042 PMC6115211

[28] Conrado DJ Karlsson MO Romero K Sarr C Wilkins JJ. Open innovation: towards sharing of data, models and workflows. Eur J Pharm Sci. (2017) 109S:S65–71. 10.1016/j.ejps.2017.06.035 28684136

[29] Wilhelm EE Oster E Shoulson I. Approaches and costs for sharing clinical research data. JAMA. (2014) 311:1201–02. 10.1001/jama.2014.850 24556937

[30] Berner E. Clinical Decision Support Systems. Theory and Practice. Health Informatics Series. New York, NY: Springer (2007).

[31] Faviez C Chen X Garcelon N Neuraz A Knebelmann B Salomon R. Diagnosis support systems for rare diseases: a scoping review. Orphanet J Rare Dis. (2020) 15:94. 10.1186/s13023-020-01374-z 32299466 PMC7164220

[32] Svenstrup DT. FindZebra – Using Machine Learning to Aid Diagnosis of Rare Diseases. DTU Computer. Kongens Lyngby: DTU Compute (2018).

[33] Long E Lin H Liu Z Wu X Wang L Jiang J et al. An artificial intelligence platform for the multihospital collaborative management of congenital cataracts. Nat Biomed Eng. (2017) 1:0024. 10.1038/s41551-016-0024

[34] Schaefer J Lehne M Schepers J Prasser F Thun S. The use of machine learning in rare diseases: a scoping review. Orphanet J Rare Dis. (2020) 15:145. 10.1186/s13023-020-01424-6 32517778 PMC7285453

[35] Brasil S Pascoal C Francisco R Dos Reis Ferreira V Videira PA Valadão AG. Artificial intelligence (AI) in rare diseases: is the future brighter? Genes. (2019) 10:978. 10.3390/genes10120978 31783696 PMC6947640

[36] Garcelon N Neuraz A Benoit V Salomon R Kracker S Suarez F. Finding patients using similarity measures in a rare diseases-oriented clinical data warehouse: Dr. Warehouse and the needle in the needle stack. J Biomed Inform. (2017) 73:51–61. 10.1016/j.jbi.2017.07.016 28754522

[37] Mitani AA Haneuse S. Small data challenges of studying rare diseases. JAMA Netw Open. (2020) 3:e201965. 10.1001/jamanetworkopen.2020.1965 32202640

[38] Li X Yu L Jin Y Fu CH Xing L Heng PH. Difficulty-aware meta-learning for rare disease diagnosis. In: Martel AL, Abolmaesumi P, Stoyanov D, et al, editors. Medical Image Computing and Computer Assisted Intervention – MICCAI 2020: 23^*rd*^ International Conference, Lima, Peru, October 4–8, 2020, Proceedings, Part I. Cham: Springer (2020). p. 357–66.

[39] Nitta H Yamazaki S Omori T Sato T. An introduction to epidemiologic and statistical methods useful in environmental epidemiology. J Epidemiol. (2010) 20:177–84. 10.2188/jea.je20100010 20431236 PMC3900838

[40] Weston J Collobert R Sinz F Bottou L Vapnik V. Inference with the Universum. In Proceedings of the 23rd International Conference on Machine Learning (ICML ‘06). New York, NY: Association for Computing Machinery (2006). p. 1009–16.

[41] Bisio F Decherchi S Gastaldo P Zunino R. Inductive bias for semi-supervised extreme learning machine. In: Cao J, Mao K, Cambria E, Man Z, Toh KA, editors. Proceedings of ELM-2014 Volume 1. Proceedings in Adaptation, Learning and Optimization. Cham: Springer (2015).

[42] Decherchi S Ridella S Zunino R Gastaldo P Anguita D. Using unsupervised analysis to constrain generalization bounds for support vector classifiers. IEEE Trans Neural Netw. (2010) 21:424–38. 10.1109/TNN.2009.2038695 20123572

[43] Zhuang F Qi Z Duan K Xi D Zhu Y Zhu H. A comprehensive survey on transfer learning. Proc IEEE. (2021) 109:43–76. 10.1109/JPROC.2020.3004555

[44] Wong SC Gatt A Stamatescu V McDonnell MD. Understanding Data Augmentation for Classification: When to Warp? International Conference on Digital Image Computing: Techniques and Applications (DICTA). Gold Coast (2016).

[45] Cui L Biswal S Glass LM Lever G Sun J Xiao C. CONAN: Complementary Pattern Augmentation for Rare Disease Detection. Proceedings of the AAAI Conference on Artificial Intelligence. Palo Alto, CA (2020). p. 614–21.

[46] Holzinger A. From machine learning to explainable AI. In: World Symposium on Digital Intelligence for Systems and Machines (DISA) Košice: Piscataway (2018).

[47] Zou H Hastie T. Regularization and variable selection via the elastic net. J Royal Stat Soc. (2005) 67:301–20. 10.1111/j.1467-9868.2005.00503.x

[48] Bae JM. The clinical decision analysis using decision tree. Epidemiol Health. (2014) 36:e2014025. 10.4178/epih/e2014025 25358466 PMC4251295

[49] Mordenti M Ferrari E Pedrini E Fabbri N Campanacci L Muselli M. Validation of a new multiple osteochondromas classification through switching neural networks. Am J Med Genet A. (2013) 161A:556–60. 10.1002/ajmg.a.35819 23401177

[50] Pestarino L Fiorito G Polidoro S Vineis P Cavalli A Decherchi S. On the Stability of Feature Selection in Multiomics Data. International Joint Conference on Neural Networks 2021 – IJCNN 2021 (2021).

[51] McCradden MD Joshi S Mazwi M Anderson JA. Ethical limitations of algorithmic fairness solutions in health care machine learning. Lancet Digit Health. (2020) 2:e221–3. 10.1016/S2589-7500(20)30065-0 33328054

[52] Nygren H Seppänen-Laakso T Castillo S Hyötyläinen T Orešič M. Liquid chromatography-mass spectrometry (LC-MS)-based lipidomics for studies of body fluids and tissues. Meth Mol Biol. (2011) 708:247–57. 10.1007/978-1-61737-985-7_15 21207295

[53] Rieke N Hancox J Li W Milletarì F Roth HR Albarqouni S. The future of digital health with federated learning. NPJ Digit Med. (2020) 3:119. 10.1038/s41746-020-00323-1 33015372 PMC7490367

[54] GAIA-X: A Federated Data Infrastructure for Europe. Available online at: http://www.data-infrastructure.eu/GAIAX/Navigation/EN/Home/home.html (accessed June 24, 2021).

[55] Warnat-Herresthal S Schultze H Shastry KL Manamohan S Mukherjee S Garg V. Swarm learning for decentralized and confidential clinical machine learning. Nature. (2021) 594:265–70. 10.1038/s41586-021-03583-3 34040261 PMC8189907

